# The Role of Freeze-Drying as a Multifunctional Process in Improving the Properties of Hydrogels for Medical Use

**DOI:** 10.3390/ph17111512

**Published:** 2024-11-10

**Authors:** Kacper Odziomek, Anna K. Drabczyk, Paulina Kościelniak, Patryk Konieczny, Mateusz Barczewski, Katarzyna Bialik-Wąs

**Affiliations:** 1 Cracow University of Technology, Faculty of Chemical Engineering and Technology, Department of Organic Chemistry and Technology, 24 Warszawska Street, 31155 Cracow, Poland; anna.drabczyk@pk.edu.pl; 2 Institute of Human Biology and Evolution, Faculty of Biology, Adam Mickiewicz University, 6 Uniwersytetu Poznanskiego Street, 61614 Poznan, Poland; paulina.koscielniak@amu.edu.pl (P.K.); patryk.konieczny@amu.edu.pl (P.K.); 3 Institute of Materials Technology, Faculty of Mechanical Engineering, Poznan University of Technology, 3 Piotrowo Street, 61138 Poznan, Poland; mateusz.barczewski@put.poznan.pl; 4 Cracow University of Technology, Faculty of Chemical Engineering and Technology, Department of Chemistry and Technology of Polymers, 24 Warszawska Street, 31155 Cracow, Poland

**Keywords:** freeze-drying, hydrogels, drug delivery, release studies, wound healing, skin diseases, salicylic acid, fluocinolone acetonide

## Abstract

**Background/Objectives:** Freeze-drying is a dehydration method that extends the shelf life and stability of drugs, vaccines, and biologics. Recently, its role has expanded beyond preservation to improve novel pharmaceuticals and their carriers, such as hydrogels, which are widely studied for both drug delivery and wound healing. The main aim of this study was to explore the multifunctional role of freeze-drying in improving the physicochemical properties of sodium alginate/poly(vinyl alcohol)-based hydrogels for medical applications. **Methods:** The base matrix and hydrogels containing a nanocarrier-drug system, were prepared by chemical cross-linking and then freeze-dried for 24 h at −53 °C under 0.2 mBa. Key analyses included determination of gel fraction, swelling ratio, FT-IR, SEM, TG/DTG, in vitro drug release and kinetics, and cytotoxicity assessment. **Results:** Freeze-drying caused an increase in the gel fraction of the hydrogel with the dual drug delivery system from 55 ± 1.6% to 72 ± 0.5%. Swelling ability was pH-dependent and remained in the same range (175–282%). Thermogravimetric analysis showed that freeze-dried hydrogels exhibited higher thermal stability than their non-freeze-dried equivalents. The temperature at 10% weight loss increased from 194.0 °C to 198.9 °C for the freeze-dried drug-loaded matrix, and from 188.4 °C to 203.1 °C for the freeze-dried drug-free matrix. The average pore size of the freeze-dried hydrogels was in the range of 1.07 µm ± 0.54 to 1.74 µm ± 0.92. In vitro drug release revealed that active substances were released in a controlled and prolonged way, according to the Korsmeyer–Peppas model. The cumulative amount of salicylic acid released at pH = 9.0 after 96 h was 63%, while that of fluocinolone acetonide reached 73%. Both hydrogels were non-toxic to human fibroblast cells, maintaining over 90% cell viability after 48 h of incubation. **Conclusions:** The results show a high potential for commercialisation of the obtained hydrogels as medical dressings.

## 1. Introduction

Freeze-drying, also known as lyophilisation, is a dehydration method used for the preservation of perishable products, additionally making them more convenient for storage. This method consists of freezing the product, reducing the pressure, and then removing the frozen water through sublimation, in which water transitions from solid directly to vapour without passing through the liquid phase [1]. Extending shelf life while at the same time maintaining the original properties of products is the reason why freeze-drying is commonly used in biomedicine. This includes the development of proteins, peptides, antibiotics, vaccines, and liposomes [2,3,4,5]. Freeze-drying is also increasingly employed in the preparation of advanced drug delivery systems, such as polymeric nanoparticles, liposomal formulations, and microspheres [6,7,8], as well as highly porous three-dimensional scaffolds for tissue engineering [9,10,11]. Hydrogels have also been studied in order to extend their application possibilities. These polymeric structures are three-dimensional, hydrophilic networks that have the ability to absorb and retain large amounts of water or biological fluids [12]. In most studies on hydrogel materials, freeze-drying is considered an integral part of the hydrogel preparation, combining both cross-linking and dehydration in a single process. For example, Autissier et al. described a novel combined freeze-drying/cross-linking method with biomacromolecules that induces pore formation in polysaccharide-based hydrogels [13]. Thus, the traditional approach focusses on the dual role of freeze-drying in creating the hydrogel network and enhancing its porosity as a result of the nucleation of ice [14], which limits their application due to frequent worsening mechanical parameters [15], such as gel fraction, maximum strength, and elongation at break [16].

To prevent damage caused by ice crystal formation during freezing, and to preserve the structure and function of both the hydrogel and any encapsulated active substances, lyoprotectants and cryoprotectants, such as sugars, polyols, and amino acids, are used [17]. Some of the most popular protectants include trehalose, sucrose, mannitol, and glycerin, which are well documented for their ability to prevent the denaturation of proteins and maintain the structure of biomaterials during freeze-drying [18]. In the context of hydrogel dressings, glycerin is important as it increases the uptake of active substances by the skin and moisturises it [19]. Interestingly, sodium alginate, a natural polysaccharide derived from brown seaweed, is also included in natural origin excipients used in the freeze-drying [18,20]. However, while lyo- and cryoprotectants offer significant benefits, they can also have unintended negative effects on the properties of hydrogels, such as reduced glass transition temperature [18]. Therefore, their concentration, type, and impact on the material’s properties must be carefully considered.

In addition to the use of lyo- and cryoprotectants, it would also be appropriate to consider the use of polymers that could reduce the previously mentioned negative effects of freeze-drying, like decreased cross-linking degree. For instance, poly(vinyl alcohol) is a great example of a polymer, which can be crosslinked based on the freeze-drying, as it has the interesting ability to form crystalline regions during the freezing phase, which allows to cross-link of poly(vinyl alcohol) without using of chemical agents. Thus, repeated freeze-thaw cycles cause phase separation and crystallisation of its chains [21,22,23]. Furthermore, it is non-toxic, biocompatible, and has good thermal and mechanical properties [24]. Its undesired clinical properties, which limit its applicability, have been improved by the development of hybrid hydrogels using sodium alginate, an additional biopolymer [25].

In fact, freeze-drying is not usually used after synthesis when the hydrogel has already been cross-linked through other mechanisms, such as chemical methods, and is already air-dried. For this reason, our research focuses on freeze-drying as a multifunctional process by combining two different methods of cross-linking and drying. Moreover, it is important to compose a hydrogel matrix with dual-action components, which could both improve its properties and protect it from the negative effects of freeze-drying. Thus, an sodium alginate/poly(vinyl alcohol)-based hydrogel and its nanocarrier-drug system-loaded equivalent [26] were selected for this study. The positive impact of poly(vinyl alcohol) and glycerin on the hydrogels was confirmed in the previous research [25,27,28].

The distinguishing feature of this study is the use of freeze-drying, an unconventional low-temperature dehydration method that was tested for the above-mentioned formulation for the first time and proved to have a positive effect on it. Moreover, the use of freeze-drying as a cross-linking method confirms its multifunctionality and emphasises the uniqueness of the investigation. The positive impact of freeze-drying on the properties of sodium alginate/poly(vinyl alcohol)-based hydrogels was confirmed by the determination of gel fraction, swelling ratio, FT-IR, SEM, TG/DTG, in vitro release profile and kinetics analyses, and cytotoxicity assessment.

## 2. Results and Discussion

### 2.1. Gel Fraction Analysis

Gel fraction (%GF) is the percentage of the hydrogel matrix that is left after soluble or leachable components are removed by immersion in distilled water. This measurement gives information on the density of cross-links and the stability of the hydrogel network. As a result, a hydrogel that has a higher gel fraction shows a greater degree of cross-linking. The gel fractions of hydrogels before and after freeze-drying are presented in Table 1.

Our research assessed the gel fraction of freeze-dried as well as non-freeze-dried hydrogel samples, as depicted in Table 1. The non-freeze-dried hydrogel matrix containing a dual drug delivery system based on a thermosensitive nanocarrier (M-SA + FA) presented a gel fraction of 55% ± 1.6, the lowest among all samples. On the other hand, the freeze-dried hydrogel with the dual drug delivery system (F-D-M-SA + FA) showed the highest gel fraction at 72% ± 0.5, suggesting a stronger and more stable network structure. The base drug-free hydrogel sample (M) was tested in the previous study on sodium alginate/poly(vinyl alcohol)-based hydrogels [27] and had a gel fraction of 63.4% ± 1.8, very similar to its freeze-dried equivalent (F-D-M), which displayed a gel fraction of 61% ± 0.8.

The minimum difference in gel fraction between the base hydrogels (M and F-D-M) is within the range of measurement errors and suggests that the freeze-drying process does not have a major impact on their cross-linking density. However, a significant increase in gel fraction value for the F-D-M-SA + FA sample implies that freeze-drying enhances the cross-linking density of the hydrogel matrix containing a nanocarrier-drug system. This is very likely to be associated with additional cross-linking of poly(vinyl alcohol) in the presence of a nanocarrier-drug system during the freezing phase. Physical cross-linking of poly(vinyl alcohol) has been observed in the literature, particularly in the freezing-thawing (F-T) method, introduced by Peppas in 1975 [21]. This method is used in the preparation of poly(vinyl alcohol)-based hydrogels, during which phase separation and crystallisation of the poly(vinyl alcohol) chains occur [22], which likely contributes to the observed growth of the gel fraction. The lack of a substantial change of the gel fraction for the F-D-M sample, and thus the difference in trend, indicates that the presence of the nanocarrier-drug system in the hydrogel matrix has a major impact on the freeze-drying effect. It most likely leads to more extensive phase separation and crystallisation of the poly(vinyl alcohol), as well as enhanced physical interactions between the polymer chains leading to an increased gel fraction. Interestingly, the lower gel fraction noted for the M-SA + FA sample implies that incorporation of the nanocarrier-drug system causes a decrease in gel fraction value of approximately 8% compared to the base matrix (M). Therefore, this may indicate that in the case of complex hydrogel systems containing a dual delivery system based on a thermosensitive nanocarrier, freeze-drying has an important role in both removing water from them and improving their physicochemical properties, such as gel fraction.

### 2.2. Swelling Analysis

**Figure 1:**
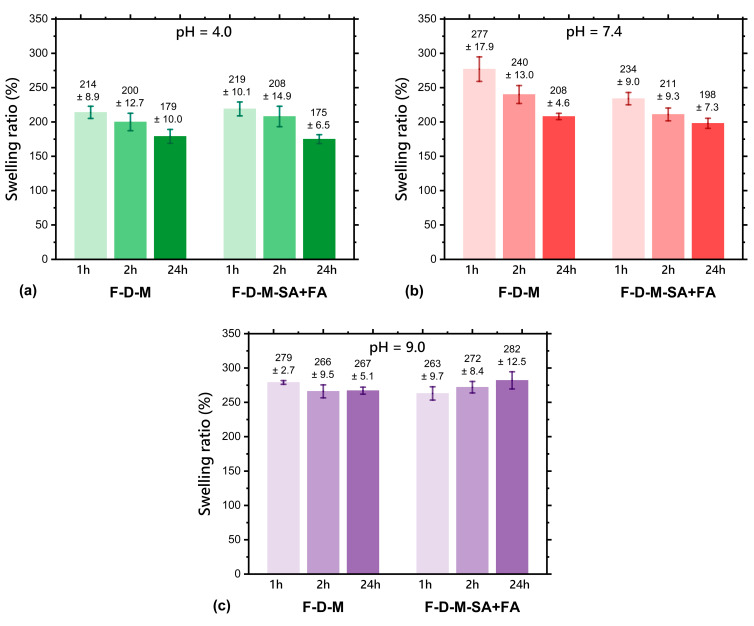
Swelling ratio (%) of the freeze-dried base matrix (F-D-M) and freeze-dried matrix containing a dual drug delivery system based on a thermosensitive nanocarrier (F-D-M-SA + FA) at 37 °C after tests in buffer solutions of pH: (**a**) 4.0; (**b**) 7.4; (**c**) 9.0. The results are presented as mean values (*n* = 3), while the bars represent ±SD.

Swelling ratio (%SR) is the percentage increase in the weight of the hydrogel due to water absorption relative to its initial dry weight. Basically, it describes the extent to which a hydrogel can absorb water or a biological fluid. Therefore, it is related to the hydrogel’s porosity, hydrophilicity, and gel fraction. The swelling ratios of hydrogels after freeze-drying are presented in Figure 1.

The swelling ratios of the freeze-dried base hydrogel matrix (F-D-M) in buffer solutions at pH = 4.0, 7.4, and 9.0 were in the ranges of 179–214%, 208–277%, and 266–279%, respectively. The swelling ratios of the freeze-dried hydrogel with the dual drug delivery system based on a thermosensitive nanocarrier (F-D-M-SA + FA) in buffer solutions at pH = 4.0, 7.4, and 9.0 were in the ranges of 175–219%, 198–234%, and 263–282%, respectively. According to the data presented in Figure 1, pH dependence on the %SR value was observed. Specifically, the higher the pH of the buffer solution, the higher the value of the swelling ratio for both freeze-dried hydrogel samples. This phenomenon was already observed in the literature [29,30] and is related to the ionic strength of buffer solution. Usually, anionic hydrogels demonstrate an increased swelling ratio in environments with a higher pH (up to a value of 9.0) because the carboxylate groups are ionised, leading to greater electrostatic repulsion and thus better water absorption. On the other hand, an acidic environment results in the protonation of carboxylate groups, resulting in the formation of neutral COOH groups. Therefore, it reduces the electrostatic repulsion between anionic groups, leading to a decreased swelling ratio [31]. In our previous research [26,32], a noticeable decrease of swelling ratio in PBS (pH = 7.4) of the hydrogel matrix after incorporating the nanocarrier-drug system, especially after one hour, was observed, similarly to results obtained after freeze-drying (Figure 1b). However, much smaller variations were observed at pH 9.0 (Figure 1c), and at pH 4.0 (Figure 1a), they did not exceed a few percentage units. Formerly, it was also recorded that introducing a nanocarrier-drug system allows a faster achievement of the equilibrium between the environment (pH = 7.4) and hydrogel sample. After freeze-drying, this correlation still occurs and can be seen in Figure 1b. Nevertheless, the fastest equilibrium achievement was noted at pH = 9.0. Overall, freeze-drying does not cause any significant changes in swelling ratios of hydrogel matrices (F-D-M and F-D-M-SA + FA).

### 2.3. FT-IR Analysis

**Figure 2:**
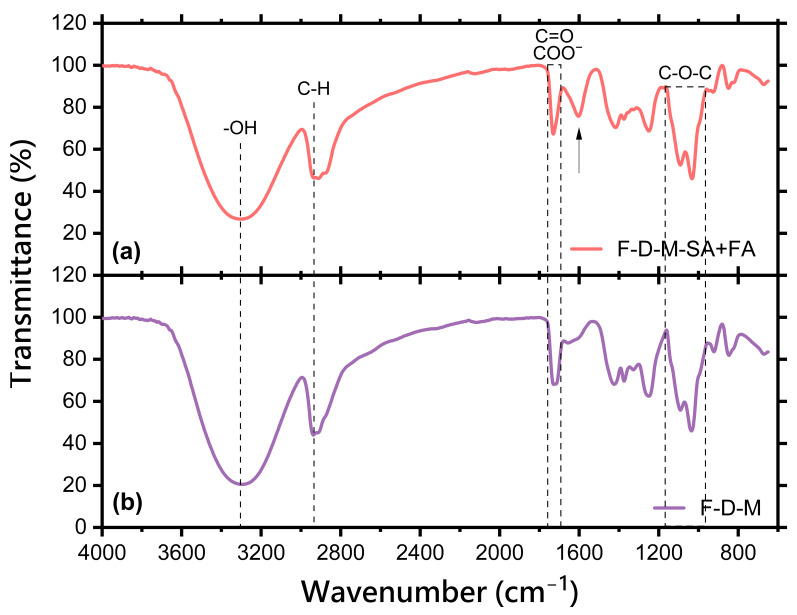
FT-IR spectra of the: (**a**) freeze-dried matrix containing a dual drug delivery system based on a thermosensitive nanocarrier (F-D-M-SA + FA); (**b**) freeze-dried base matrix (F-D-M). The arrow represents an additional band at the wavenumber of ~1600 cm^−1^, which can be related to benzene ring vibration.

Fourier transform infrared spectroscopy (FT-IR) is a technique used to analyse the chemical composition of hydrogels through the identification of specific molecular bonds based on their infrared absorption. This is especially important for investigating potential structural changes in hydrogels both after modification with active substances and other used processes such as freeze-drying. The FT-IR spectra of hydrogels after freeze-drying are presented in Figure 2.

Based on the FT-IR spectra, the chemical structures of the obtained freeze-dried base matrix as well as containing salicylic acid and fluocinolone acetonide, were confirmed. The vibration of -OH bond stretching is visible very well in the range of ~3300 cm^−1^, which comes from components of the base matrix, such as poly(vinyl alcohol), sodium alginate, and aloe vera. A band with a wavenumber of ~2940 cm^−1^ was observed, which corresponds to the stretching vibrations of the C-H groups. The FT-IR spectra of all hydrogel samples present peaks in the ranges of 1720–1722 cm^−1^ and 1611–1636 cm^−1^, which are associated with C=O stretching vibrations in the PEGDA structure and COO^-^ asymmetric stretching vibrations in SA, respectively. Bands at 1245–1246 cm^−1^ and 1031–1037 cm^−1^ can be assigned to the presence of the C-O-C group derived from glycosidic bonds [28,32,33].

Interestingly, the FT-IR spectrum of the freeze-dried matrix containing the system of thermosensitive nanocarrier with drugs (F-D-M-SA + FA) shows an additional band at the wavenumber of ~1600 cm^−1^, which can be related to benzene ring vibration [34]. Considering the concentration of drugs introduced into the polymer nanocarrier system, they most likely originate from salicylic acid. Probably, it can be caused by the crystallisation processes of salicylic acid during freeze-drying. However, the presence of drug on the surface of freeze-dried hydrogels does not reduce the final properties of obtained materials.

### 2.4. SEM Analysis

**Figure 3:**
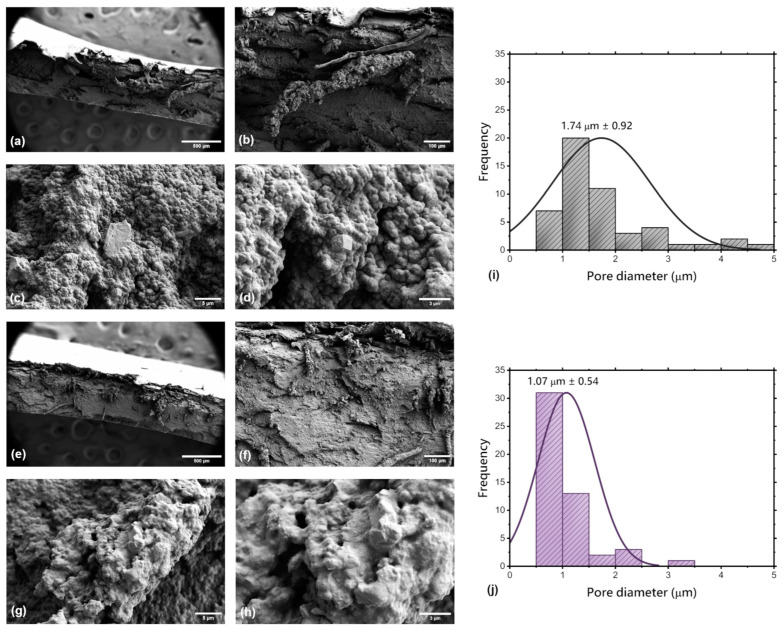
The cross-section SEM images of the freeze-dried base matrix (F-D-M) at magnifications: (**a**) 150×; (**b**) 500×; (**c**) 10,000×; (**d**) 20,000×, the cross-section SEM images of the freeze-dried matrix containing a dual drug delivery system based on a thermosensitive nanocarrier (F-D-M-SA + FA) at magnifications: (**e**) 150×; (**f**) 500×; (**g**) 10,000×; (**h**) 20,000×, and pore size distributions of samples: (**i**) F-D-M; (**j**) F-D-M-SA + FA.

In this study, SEM was used to analyse the cross-sectional morphology of the freeze-dried hydrogels, which is crucial in designing materials for tissue engineering or regenerative medicine because of its impact on cell proliferation, tissue regeneration, and the desired mechanical properties of the obtained hydrogels [35]. The cross-section SEM images, and pore size distributions of the freeze-dried hydrogels are presented in Figure 3.

Scanning Electron Microscopy (SEM) revealed that the cross-section of the freeze-dried base hydrogel matrix (F-D-M) (Figure 3a–d) is comparable to the cross-section of its non-freeze-dried equivalent (M). However, it was noticed that the structure is slightly denser and irregular. The average pore size was estimated to be 1.74 µm ± 0.92, and the range is analogous to the M sample, whose morphology was analysed in our previous research [26,27,32]. Structural integrity appears well-maintained, which suggests that the freeze-drying process does not significantly change the overall morphology. Similarly, the cross-sectional SEM images of the freeze-dried hydrogel matrix containing a dual drug delivery system based on a thermosensitive nanocarrier (F-D-M-SA + FA) (Figure 3e–h) show a structural resemblance to its non-freeze-dried equivalent (M-SA + FA). The structure is consistent, as well as for the M-SA + FA sample, which was analysed previously [26]. The dual drug delivery system still appears to be well-incorporated into the hydrogel matrix, suggesting that freeze-drying does not significantly alter the distribution of this system within the matrix. The average pore size was estimated to be 1.07 µm ± 0.54. A minor difference between pore sizes could be associated with the presence of the nanocarrier-drug system incorporated into the F-D-M-SA + FA sample, which potentially reduces the extent of empty space, limiting the formation of larger pores.

### 2.5. Thermogravimetric Analysis (TG/DTG)

**Figure 4:**
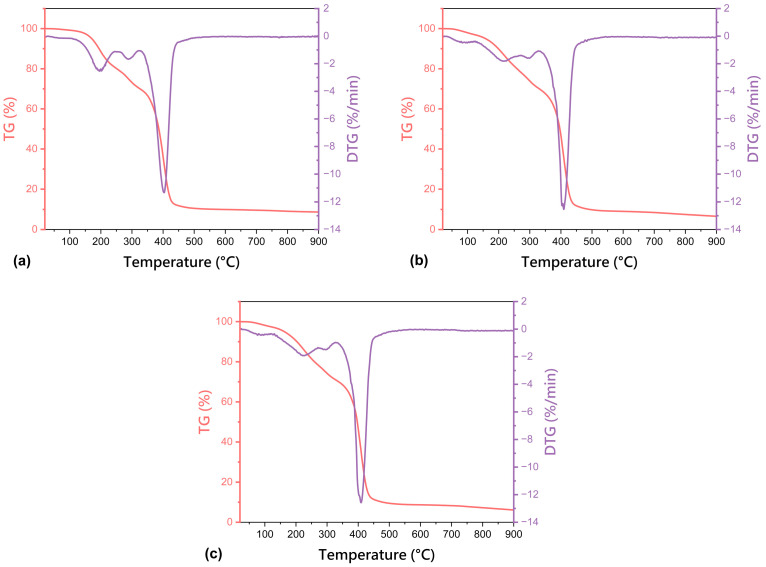
TG and DTG curves of the: (**a**) non-freeze-dried matrix containing a dual drug delivery system based on a thermosensitive nanocarrier (M-SA + FA); (**b**) freeze-dried matrix containing a dual drug delivery system based on a thermosensitive nanocarrier (F-D-M-SA + FA); (**c**) freeze-dried base matrix (F-D-M).

The thermogravimetric analysis curves (TG/DTG) of non-freeze-dried matrix containing a dual drug delivery system based on a thermosensitive nanocarrier (M-SA + FA), freeze-dried matrix containing a dual drug delivery system based on a thermosensitive nanocarrier (F-D-M-SA + FA), and freeze-dried base matrix (F-D-M) are presented in Figure 4. Moreover, the characteristic thermal parameters determined from the TG and DTG curves of tested hydrogels, i.e., temperature values at 10% and 50% mass loss (T_10%_, T_50%_), characteristic temperature values corresponding to peak DTG and residual mass at 900 °C, are compared in Table 2.

TG was used to investigate the effect of the freeze-drying process on the thermal stability of the hydrogel materials. Based on the TG and DTG curves, four different stages of thermal degradation of all analysed samples, were observed. The first step at around 40–160 °C is directly connected with the dehydration of residual water molecules trapped in the hydrogel structure. The second visible change appeared at ~200 °C, which results from the onset of degradation as well as the melting of poly(vinyl alcohol) chains [28]. It turned out that, in the case of the freeze-dried hydrogel matrix containing a dual drug system based on a thermosensitive nanocarrier (F-D-M-SA + FA) and its non-freeze-dried equivalent (M-SA + FA), the temperature at which 10% weight loss of each sample occurred increased from 194.0 °C to 198.9 °C. Importantly, a similar dependence was confirmed for the freeze-dried base matrix (F-D-M) and its non-freeze-dried equivalent (described in our previous studies [28]), because the temperature at which 10% weight loss of each sample occurred increased from 188.4 °C to 203.1 °C. The third stage was at ~300 °C, and after that, the final step was at ~400 °C, which probably comes from the degradation of the crosslinking agent, PEGDA [36].

To sum up, the freeze-drying process improved the thermal resistance of the obtained hydrogel materials because it directly impacts the crosslinking degree (higher gel fraction results (Table 1) and slightly denser structure (Figure 3)).

### 2.6. Analysis of Salicylic Acid and Fluocinolone Acetonide In Vitro Release

**Figure 5:**
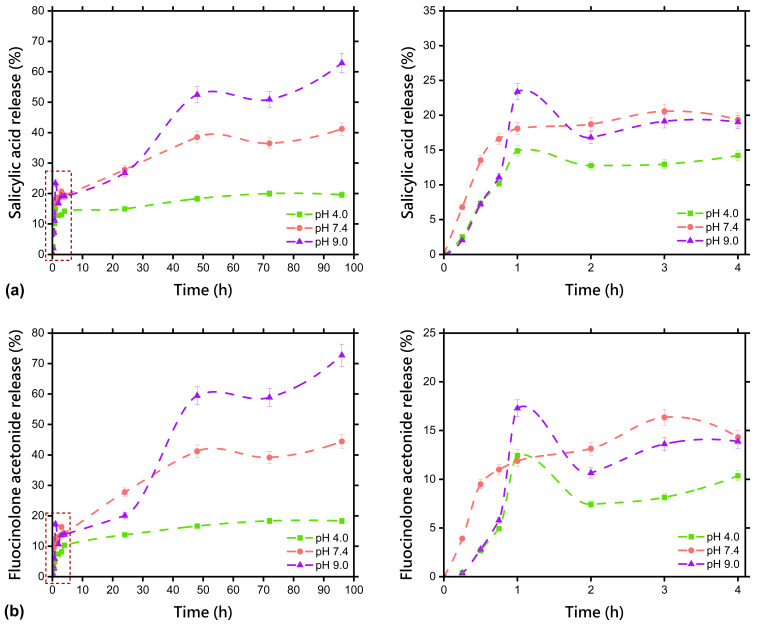
Release profile of: (**a**) salicylic acid; (**b**) fluocinolone acetonide from the freeze-dried matrix containing a dual drug delivery system based on a thermosensitive nanocarrier (F-D-M-SA + FA). The bars represent ±SD. The dotted squares represent the first phase of drugs release, which is magnified in the graphs on the right.

The drug release profile describes how active substances encapsulated in the hydrogel matrix are released over time. The release profiles of salicylic acid and fluocinolone acetonide from the freeze-dried hydrogel matrix containing a dual drug delivery system based on a thermosensitive nanocarrier (F-D-M-SA + FA) are presented in Figure 5.

In the first phase of the in vitro release of salicylic acid from the F-D-M-SA + FA sample (Figure 5a), fast release was observed, especially in acceptor solution at pH = 9.0, in which 23% of the drug was released after one hour. This phenomenon was also recorded before freeze-drying [26], however, the amount of salicylic acid released after one hour averaged 38% then. For the acceptor solution at pH = 4.0, it can be seen that release of salicylic acid occurs very similarly as before freeze-drying because the released amount after one hour is 15%. Curiously, the biggest change can be observed for the release of salicylic acid at pH = 7.4, as after three hours its released amount was 21%, which is twice as much as before freeze-drying. The fast release in the first phase may occur due to the faster diffusion of drug particles, which can be located on the surface of the hydrogel matrix, notably in the case of high drug loading [37,38,39]. Additionally, drying can also have an impact on the presence of this phenomenon because of drugs migration during the process and therefore their heterogeneous distribution in the matrix [37]. In this case, faster release may be present as a result of freeze-drying, during which a certain amount of salicylic acid probably crystallised on the surface of the hydrogel, as explained in Section 2.3. In the second phase, a prolonged release of salicylic acid (up to 96 h) occurred in tested environments, but it was observed that as the pH of the acceptor solution decreased, the release was more constant. The amounts of salicylic acid released at pH = 4.0, 7.4, and 9.0 after 96 h were 20%, 41%, and 63%, respectively. Accordingly, the released amount grew with the increase in pH value, which can be explained by the higher swelling ratio at the higher pH value of the solution (Figure 1). Importantly, an overall decrease in the release of salicylic acid was noted, specifically at pH = 4.0 and 7.4, which is likely due to an enhanced gel fraction through freeze-drying (Table 1) as well as the already mentioned swelling capacity.

In the first phase of the in vitro release of fluocinolone acetonide from the F-D-M-SA + FA sample (Figure 5b), a similar correlation was observed. Namely, a fast release in the acceptor solution at pH = 9.0, in which 17% of the drug was released after one hour, clearly less than before freeze-drying, when the value was up to 32%. In the case of the acceptor solution at pH = 4.0, the fast release is also noticeable. Similar to salicylic acid, the biggest change in release in that phase can be observed at pH = 7.4, as after three hours the released amount of fluocinolone acetonide was 16%, five times more than before freeze-drying. Prolonged release occurred in the second phase, and the noted correlation of increased constancy with a decrease in the pH of the acceptor solution was maintained. The amounts of released fluocinolone acetonide at pH = 4.0, 7.4, and 9.0 after 96 h were 18%, 44%, and 73%, respectively. Again, the amount of released drug increased with rising pH values. An overall decrease in fluocinolone acetonide release was reported, particularly at pH = 4.0. Surprisingly, an increase of 4.5% was observed at pH = 9.0.

### 2.7. Kinetic Analysis of Salicylic Acid and Fluocinolone Acetonide In Vitro Release

**Figure 6:**
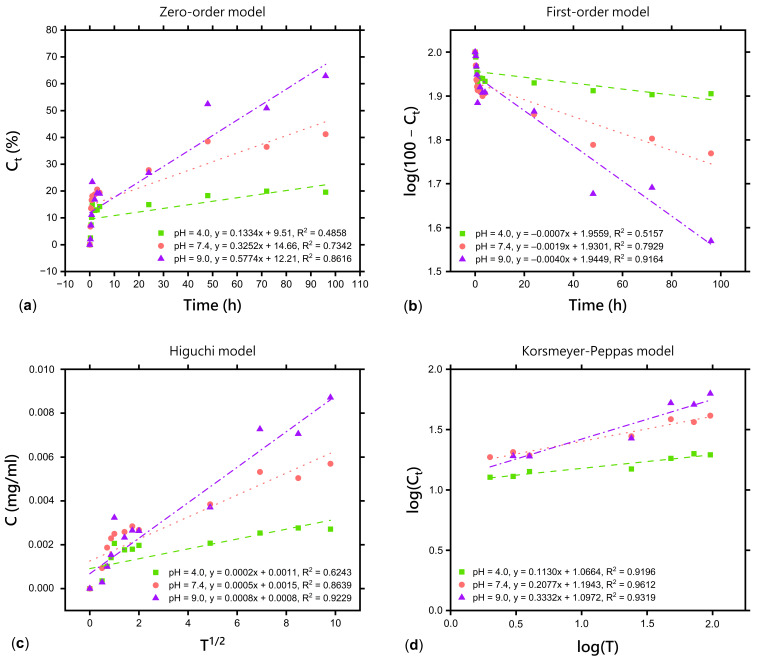
Kinetics of the salicylic acid release from freeze-dried matrix containing a dual drug delivery system based on a thermosensitive nanocarrier (F-D-M-SA + FA) according to: (**a**) zero-order model; (**b**) first-order model; (**c**) Higuchi model; (**d**) Korsmeyer–Peppas model.

**Figure 7:**
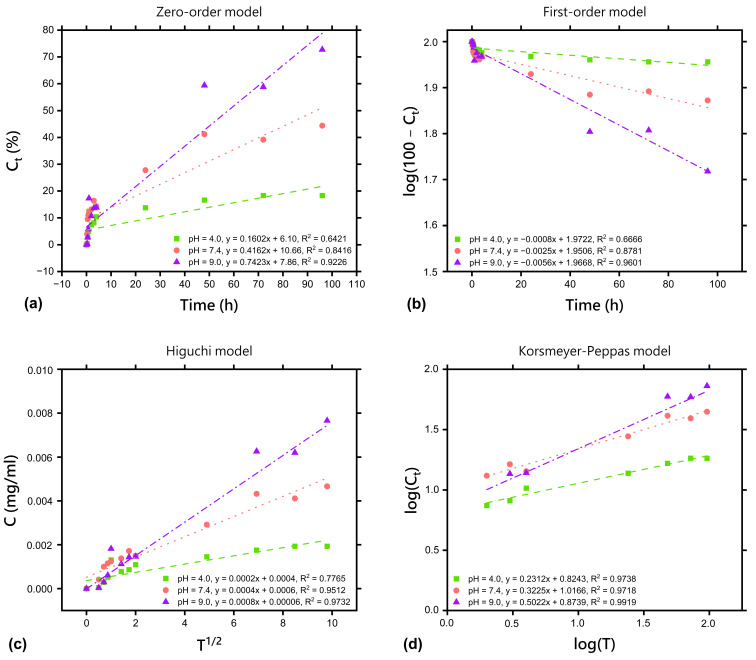
Fluocinolone acetonide release kinetics according to: (**a**) zero-order model; (**b**) first-order model; (**c**) Higuchi model; (**d**) Korsmeyer–Peppas model.

The kinetics of salicylic acid and fluocinolone acetonide release from the freeze-dried matrix containing a dual drug delivery system based on a thermosensitive nanocarrier (F-D-M-SA + FA) can be interpreted using four mathematical models: zero-order (Figure 6a and Figure 7a), first-order (Figure 6b and Figure 7b), Higuchi (Figure 6c and Figure 7c), and Korsmeyer–Peppas (Figure 6d and Figure 7d). The best-fitting kinetic model was chosen by comparison of the coefficients of determination (*R^2^*). The calculated parameters are presented in Table 3 and Table 4.

The coefficients of determination (*R*^2^_pH=4.0_ = 0.9196; *R*^2^_pH=7.4_ = 0.9612; *R*^2^_pH=9.0_ = 0.9319) presented in Table 3 confirm that the kinetics of salicylic acid release from the F-D-M-SA + FA sample is best represented by the Korsmeyer–Peppas model, the same as before freeze-drying [26]. Moreover, according to the diffusional release exponents (*n*_pH=4.0_ = 0.1130; *n*_pH=7.4_ = 0.2077; *n*_pH=9.0_ = 0.3332), it was found that release of salicylic acid from the F-D-M-SA + FA sample into acceptor solution at all used pH values follows Fickian diffusion kinetics (*n* ≤ 0.45). This type of release mechanism indicates a usual drug diffusion by chemical potential gradient, with no impact of erosion (relaxation) or swelling of the polymer matrix [40,41,42,43]. Interestingly, a noticeable difference in diffusional release exponent at pH = 7.4 was observed. In our previous study [26], release of salicylic acid from the M-SA + FA sample into acceptor solution at pH = 7.4 followed anomalous (non-Fickian) transport, which means it was controlled by more than one process. Therefore, the difference in the transport mechanism of salicylic acid at this pH value is most likely a result of freeze-drying, which increases the gel fraction of the F-D-M-SA + FA sample. As we have already mentioned, the gel fraction refers to the cross-linked part of the hydrogel, which is not soluble in water. Hence, in the F-D-M-SA + FA sample, polymer chains are more closely bound and less susceptible to relaxation, so the release of salicylic acid is mainly controlled by diffusion, which is observed by a decrease in diffusional release exponents. Fickian diffusion provides a controlled, predictable, and steady release of the drug over time [44], which is often desired in long-term therapies. Thus, freeze-drying has no negative effect on the release kinetics of salicylic acid from the F-D-M-SA + FA sample into acceptor solution at pH = 7.4 and can be used to modify the release kinetics when required.

The coefficients of determination (*R*^2^_pH=4.0_ = 0.9738; *R*^2^_pH=7.4_ = 0.9718; *R*^2^_pH=9.0_ = 0.9919) presented in Table 4 confirm that the kinetics of fluocinolone acetonide release from the F-D-M-SA + FA sample can be described by the Korsmeyer–Peppas model, as previously. According to the diffusional release exponents of the Korsmeyer–Peppas model presented in Table 4 (*n*_pH=4.0_ = 0.2312; *n*_pH=7.4_ = 0.3225; *n*_pH=9.0_ = 0.5022), it may be noted that release of fluocinolone acetonide at pH = 4.0 still follows Fickian diffusion kinetics (*n* ≤ 0.45). However, a difference in *n* value at pH = 7.4 is observed, similarly to salicylic acid release, the mechanism of which changed from anomalous (non-Fickian) to Fickian diffusion. Interestingly, a change of mechanism from Fickian diffusion to anomalous at pH = 9.0 was recorded. It is worth mentioning that all calculated coefficients of determination (*R^2^*) for the release of fluocinolone acetonide at pH = 9.0 are high and their values are close to each other.

In fact, physiological conditions are much more complex than conditions controlled by buffer solutions or simulated body fluids under in vitro research and may affect pharmacokinetics [45]. Therefore, to develop further studies on the impact of freeze-drying the hydrogels on salicylic acid and fluocinolone acetonide release kinetics, in vivo release analysis should be considered in the future.

### 2.8. In Vitro Biological Analysis

**Figure 8:**
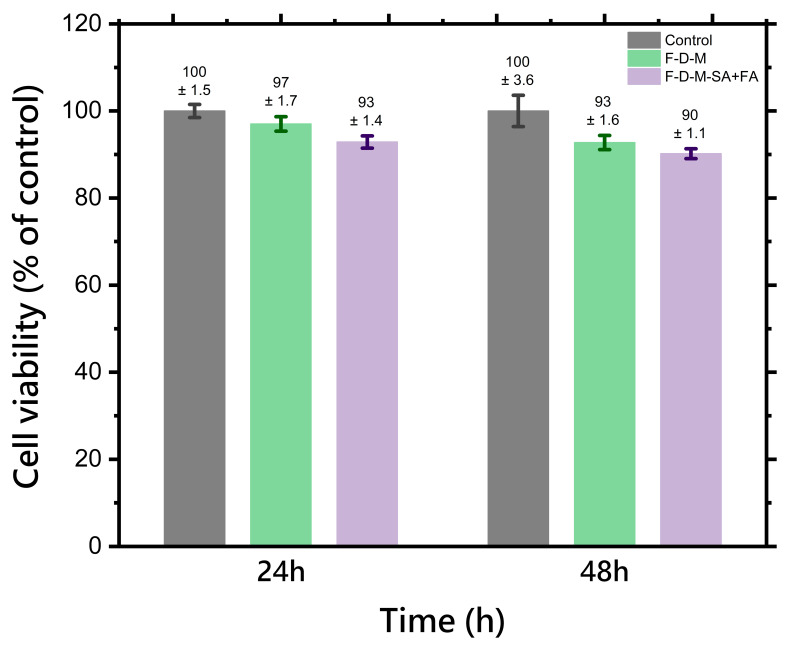
The cell viability in the presence of freeze-dried matrix containing a dual drug delivery system based on a thermosensitive nanocarrier (F-D-M-SA + FA) and freeze-dried base matrix (F-D-M). The results are presented as mean values (*n* = 3), while the bars represent ±SD.

The cytotoxicity assessment of potential biomaterials for medical applications is crucial. The dependence of cell viability on time is presented in Figure 8.

The cytotoxicity results indicated clearly that freeze-dried matrix containing a dual drug delivery system based on a thermosensitive nanocarrier (F-D-M-SA + FA) and freeze-dried base matrix (F-D-M) are non-toxic and safe for human fibroblast cells. After 48 h of incubation, all hydrogel samples were characterised by cell viability at a level above 90%. Positive results of in vitro biological studies enable further development of hybrid hydrogels as innovative wound dressing materials.

## 3. Materials and Methods

### 3.1. Materials

Table 5 contains a list of all substrates used in this research, along with details such as molecular weight, name of the producer, and purity degree.

### 3.2. Synthesis of the Empty Thermosensitive Nanocarrier (T)

The empty thermosensitive nanocarrier was prepared by constant stirring of 0.5% solution of gum arabic, N-isopropylacrylamide, and N,N′-methylenebisacrylamide, heated up to 70 °C in a glycerin bath under an inert gas atmosphere. Subsequently, ammonium persulfate was introduced, and the reaction mixture was heated up to 80 °C for 4 h. Finally, the thermosensitive nanocarrier was purified by dialysis with a cellulose membrane (MWCO = 14,000 Da). All details on synthesis were provided in previous research and literature [26,32,46,47].

### 3.3. Encapsulation of the Salicylic Acid and Fluocinolone Acetonide into the Thermosensitive Nanocarrier (T-SA + FA)

The encapsulation procedure involved mixing purified thermosensitive nanocarrier (T) (average size: 118 nm) [32] with salicylic acid and fluocinolone acetonide, both dissolved in ethyl alcohol, at a consistent stirring speed. Then, a freeze-drying process was used to create a dual drug delivery system utilising a thermosensitive nanocarrier. All details about the encapsulation of salicylic acid and fluocinolone acetonide into the thermosensitive nanocarrier T-SA + FA (369 nm) [26] were presented in the previous study [48].

### 3.4. Preparation of the Hydrogels Containing a Dual Drug Delivery System Based on a Thermosensitive Nanocarrier

The hydrogels’ synthesis was primarily based on the method previously described [27,28] through conventional chemical cross-linking using a 2% (*w*/*v*) solution of sodium alginate, a 5% (*w*/*v*) solution of poly(vinyl alcohol), Aloe vera lyophilisate, glycerin (1.7% *v*/*v*), a dual drug delivery system based on a thermosensitive nanocarrier (T-SA + FA), a 1% (*w*/*v*) solution of ammonium persulfate as an initiator, and poly(ethylene glycol) diacrylate (PEGDA, M_n_ = 700 g/mol) (7.5% *v*/*v*) as a cross-linking agent [26,32,48,49,50].

### 3.5. Freeze-Drying of the Hydrogels

**Figure 9:**
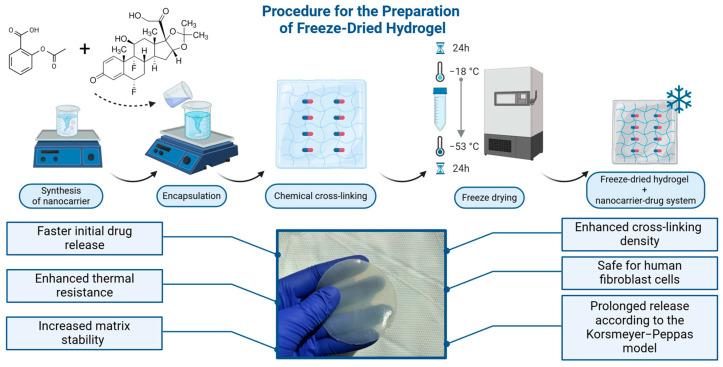
The preparation scheme of the freeze-dried hydrogels.

The process started with a pre-freezing phase, in which hydrogel samples were frozen at −18 °C for 24 h. After pre-freezing, a freeze-drying phase was carried out by placing the frozen hydrogel samples in the ChristAlpha 1–2 LD Plus freeze-dryer (Martin Christ, Osterode am Harz, Germany) at −53 °C under 0.2 mBa. The applied temperature and vacuum conditions allowed sublimation of the ice directly from solid to vapour without passing through a liquid phase, maintaining the original hydrogel matrix structure. The freeze-drying was carried out for 24 h to completely remove unbound water. Ultimately, hydrogels were obtained: a freeze-dried hydrogel matrix containing a dual drug delivery system based on a thermosensitive nanocarrier (F-D-M-SA + FA) and a freeze-dried base drug-free hydrogel (F-D-M) were prepared as the reference samples. A scheme for the preparation of freeze-dried hydrogel materials is presented in Figure 9.

### 3.6. Determination of Gel Fraction

The freeze-dried hydrogels (F-D-M-SA + FA and F-D-M) as well as non-freeze-dried hydrogels (M-SA + FA and M) [26] were cut into 10 × 10 mm pieces, then dried at 40 °C for 24 h and weighed (W_0_). Afterward, they were immersed in distilled water for 48 h until reaching a stable, swollen weight to remove any leachable or soluble parts. Next, the hydrogels were dried at 40 °C for 24 h and weighed again (W_E_). The gel fraction (%GF) was determined using the following Equation (1):

%GF = (W_E_/W_0_) ∙ 100% (1)

### 3.7. Determination of Swelling Ratio

The %SR was determined by immersing previously weighed (W_D_) 10 × 10 mm freeze-dried hydrogel samples (F-D-M-SA + FA and F-D-M) in an excess of buffer solutions (pH = 4.0, 7.4, and 9.0) at 37 °C. The swollen samples were taken out and weighed (W_S_) after 1, 2, and 24 h. The water absorption of each hydrogel sample was calculated using the following Equation (2):

%SR = (W_S_ − W_D_)/W_D_ ∙ 100% (2)

### 3.8. Attenuated Total Reflectance-Fourier Transform Infrared Spectroscopy (ATR-FTIR)

To identify the chemical structure of freeze-dried hydrogels (F-D-M-SA + FA and F-D-M), attenuated total reflectance-Fourier transform infrared spectroscopy was performed using a Thermo Scientific Nicolet iS5 FT-IR spectrometer (Thermo Fisher Scientific, Waltham, MA, USA) equipped with an iD7 ATR accessory in the range of 4000–400 cm^−1^.

### 3.9. Scanning Electron Microscopy (SEM)

The Scanning Electron Microscope Apreo 2 S LoVac (Thermo Fisher Scientific, Waltham, MA, USA) was used to analyse the surface morphology and cross-section of freeze-dried hydrogels (F-D-M-SA + FA and F-D-M), fitted with an UltraDry detector and the Octane Elect detector (EDAX Ametek GmbH, Weiterstadt, Germany). Two detectors were employed to observe the specimen: the Everhart–Thornley (ETD) detector and the T1 detector (BSE). The samples were sputter-coated with a 5 nm layer of gold in an argon atmosphere. The analysis was performed under high vacuum conditions.

### 3.10. Thermogravimetric Analysis (TGA)

Thermogravimetric analysis was carried out with a TG 209 F1 Libra instrument (Netzsch, Selb, Germany). Hydrogel samples with a mass of 8.0 ± 0.1 mg were placed in Al_2_O_3_ crucibles and, after that, they were heated from 25 to 900 °C at a heating rate of 10 °C · min^−1^ under a nitrogen atmosphere.

### 3.11. In Vitro Release of Salicylic Acid and Fluocinolone Acetonide

To investigate the release profiles of salicylic acid and fluocinolone acetonide from the freeze-dried hydrogel containing a dual drug delivery system based on a thermosensitive nanocarrier (F-D-M-SA + FA), an in vitro drug release study was conducted by the previously described static method [26]. Firstly, the entire hydrogel matrix was weighed. Next, three samples weighing approximately 250 mg each were placed in the regenerated cellulose (RC) dialysis membranes Spectra/Por^®^ MWCO = 6000–8000 Da from Carl Roth^®^Company (Karlsruhe, Germany). Each dialysis membrane containing a sample was inserted in a thermostatic chamber with 250 mL of acceptor solution: 2% (*w*/*w*) ethyl alcohol in buffer solution with pH values of, respectively, 4.0, 7.4, and 9.0. The fluids were stirred at constant speed at 37 °C. At appropriate time intervals, 2 mL of each solution was taken out, and an equal amount of fresh acceptor solutions was put back into the chamber. The experiment was carried out over a period of 96 h. Concentrations of the salicylic acid and fluocinolone acetonide released into the fluids were measured using UV-VIS spectroscopy (Evolution 220, Thermo Fisher Scientific, Waltham, MA, USA) at wavelengths of 295 nm and 243 nm, respectively.

### 3.12. In Vitro Release Kinetics of Salicylic Acid and Fluocinolone Acetonide

In order to study salicylic acid and fluocinolone acetonide release kinetics, four mathematical models: zero-order (Equation (3)), first-order (Equation (4)), Higuchi (Equation (5)), and Korsemeyer–Peppas (Equation (6)) were analysed by linear regression of the curves. The mathematical model with the highest coefficient of determination (*R*^2^) was chosen as the most accurate in representing the release kinetics [51]. To study the mechanism of transport of the drugs through the freeze-dried hydrogel matrix, diffusional release exponents (*n*) were calculated from the slope of the straight line of that model.

*C_t_* = *K*_0_ ∙ *t* (3)

log(100 − *C_t_*) = (−*K*_1_ ∙ *t*)/2.303 (4)

*C_t_* = *K_H_* ∙ √*t* (5)

log*C_t_* = log*K_KP_* + *n* ∙ log*t* (6)

*C_t_*—amount of drug released in time
*K*_0_—zero-order kinetic constant
*K*_1_—first-order kinetic constant
*K_H_*—Higuchi kinetic constant
*K_KP_*—Korsmeyer-Peppas kinetic constant
*n*—diffusional release exponent
*t*—time

### 3.13. Cytotoxicity Test

Primary human fibroblast cells (GM07452, Coriell Institute for Medical Research) were cultured in Minimum Essential Medium (MEM) supplemented with 10% Fetal Bovine Serum (FBS), 1% antibiotic, and 1% amino acids. The cells were incubated at 37 °C in a 5% CO_2_ incubator. Cells were plated 3 days prior to cytotoxicity testing to achieve over 90% confluence. Freeze-dried hydrogels (F-D-M and F-D-M-SA + FA) were soaked in sterile ddH_2_O and transferred to the bottom of a black 96-well plate. 100 µL of cells suspended in medium were added to the plate, including each type of hydrogel and control wells without hydrogel, in three biological replicates per variant. Cytotoxicity was evaluated using the CytoTox-Fluor™ assay following the manufacturer’s protocol, measuring absorbance at 480 nm (excitation wavelength) and emission at 525 nm (emission wavelength) after 1 h of incubation. Fluorescence was measured using a Tecan Infinite M200 PRO fluorescence reader (Durham, NC, USA) after 24 and 48 h of incubation for cells grown with and without hydrogels. The fluorescence signal from samples containing F-D-M or F-D-M-SA + FA hydrogels was compared to the control, and the results were expressed as a percentage increase over control cells (set at 100%).

### 3.14. Statistical Analysis

All data concerning the gel fraction, swelling ratio, drug release, and kinetics analyses, as well as cytotoxicity tests, were presented as a mean of three different experiments ± SD. Differences between the calculated means of each individual group were determined by one-way ANOVA tests using the statistical software Statistica Version 12 from StatSoft Company (Cracow, Poland). The value of *p* < 0.05 was considered statistically significant.

## 4. Conclusions

In this study, the effect of freeze-drying on sodium alginate/poly(vinyl alcohol)-based hydrogels, both with and without a nanocarrier-drug system, was comprehensively investigated. The results proved that freeze-drying significantly enhanced the cross-linking density of the hydrogel containing a dual drug delivery system based on a thermosensitive nanocarrier (F-D-M-SA + FA), as evidenced by the increase of the gel fraction. Probably, it is caused by the additional crystallisation of poly(vinyl alcohol) during the freezing process, which contributes to stronger network formation and thus, better stability of the matrix. The TG/DTG results exhibited that all freeze-dried hydrogel samples had better thermal resistance compared to non-freeze-dried samples. Swelling behaviour was found to be pH-dependent, with the highest swelling ratios observed in a more alkaline environment (pH 9.0). The chemical structure of the freeze-dried hydrogels was confirmed based on the FT-IR spectra analysis. Interestingly, in the case of the F-D-M-SA + FA sample, the characteristic band was observed at a wavenumber of ~1600 cm^−1^, which can be related to the benzene ring vibration presenting in the salicylic acid. Fortunately, it does not impact biological properties and drug release. The cytotoxicity tests showed that all analysed hydrogel materials are non-toxic. Additionally, SEM images revealed a consistent microstructure in both hydrogel samples, suggesting that the freeze-drying process influences the morphological properties slightly. A faster release of drugs was observed in the initial phase, especially at the higher pH level, but an overall reduction in the cumulative drug release was noted, likely due to the enhanced cross-linking. The release kinetics of both salicylic acid and fluocinolone acetonide showed some differences across pH levels. At pH 7.4, the release mechanism of both drugs shifted from anomalous (non-Fickian) to Fickian diffusion, indicating a more diffusion-controlled release process. Interestingly, at pH 9.0, the release mechanism of fluocinolone acetonide changed from Fickian to anomalous diffusion, reflecting a more complex release behaviour in the alkaline environment.

In conclusion, freeze-drying proved to be an effective method for improving the properties of sodium alginate/poly(vinyl alcohol)-based hydrogel with a nanocarrier-drug system while maintaining favourable active substances release profiles.

## Figures and Tables

**Table 1:** Gel fraction (%) of the freeze-dried base matrix (F-D-M), freeze-dried (F-D-M-SA + FA), and non-freeze-dried (M-SA + FA) matrix containing a dual drug delivery system based on a thermosensitive nanocarrier. The results are presented as mean values (*n* = 3) with ±SD.

Sample	GF (%) ± SD
F-D-M-SA + FA	72 ± 0.5
F-D-M	61 ± 0.8
M-SA + FA	55 ± 1.6

**Table 2:** Thermal degradation profiles of analysed hydrogels.

**Hydrogel Sample**	**T_10_ (°C)**	**T_50_ (°C)**	**T_f_ (°C)**	**DTG Peaks (°C; %/min)**	**Residual Mass (%)**
M-SA + FA	194.0	386.4	402.0	75.5; −0.25 197.1; −2.55 289.3; −1.67 402.0; −11.35	8.72
F-D-M-SA + FA	198.9	396.7	409.3	99.3; −0.49 215.3; −1.81 298.9; −1.59 409.3; −12.54	6.62
F-D-M	203.1	397.1	409.0	76.7; −0.44 226.7; −1.92 295.0; −1.49 409.0; −12.58	6.15

Temperatures at which 10% and 50% weight loss was recorded by TG at heating rate 10 °C·min^−1^ in N_2_ atmosphere, respectively.

**Table 3:** Parameters determined for mathematical models describing the kinetics of salicylic acid release from freeze-dried matrix containing a dual drug delivery system based on a thermosensitive nanocarrier (F-D-M-SA + FA).

Mathematical Model	Linear Regression	Model Parameters	Parameter Value Calculated for a Given Sample		
			pH = 4.0	pH = 7.4	pH = 9.0
Zero-order model	*C_t_ = K_0_ ∙ t*	*R*^2^ *K*_0_ (mg/h)	0.4858	0.7342	0.8616
			0.1334	0.3252	0.5774
First-order model	log(100 − *C_t_*) = (−*K_1_ ∙ t*)/2.303	*R*^2^ *K*_1_ (1/h)	0.5157	0.7929	0.9164
			0.0016	0.0044	0.0092
Higuchi model	*C_t_* = *K_H_* ∙ *√t*	*R*^2^ *K_H_* (mg/h^1/2^)	0.6243	0.8639	0.9229
			0.0002	0.0005	0.0008
Korsmeyer–Peppas model	log*C_t_* = log*K_KP_* + *n* ∙ log*t*	*R*^2^ *K_KP_* (h^−n^) *n*	0.9196	0.9612	0.9319
			11.65	15.64	12.51
			0.1130	0.2077	0.3332

**Table 4:** Parameters determined for mathematical models describing the kinetics of fluocinolone acetonide release from freeze-dried matrix containing a dual drug delivery system based on a thermosensitive nanocarrier (F-D-M-SA + FA).

Mathematical Model	Linear Regression	Model Parameters	Parameter Value Calculated for a Given Sample		
			pH = 4.0	pH = 7.4	pH = 9.0
Zero-order model	*C_t_* = *K*_0_ ∙ *t*	*R*^2^ *K*_0_ (mg/h)	0.6421	0.8416	0.9226
			0.1602	0.4162	0.7423
First-order model	log(100 − *C_t_*) = (−*K*_1_ ∙ *t*)/2.303	*R*^2^ *K*_1_ (1/h)	0.6666	0.8781	0.9601
			0.0018	0.0058	0.0129
Higuchi model	*C_t_* = *K_H_* ∙ √*t*	*R*^2^ *K_H_* (mg/h^1/2^)	0.7765	0.9512	0.9732
			0.0002	0.0004	0.0008
Korsmeyer–Peppas model	log*C_t_* = log*K_KP_* + *n* ∙ log*t*	*R*^2^ *K_KP_* (h^−n^) *n*	0.9738	0.9718	0.9919
			6.67	10.39	7.48
			0.2312	0.3225	0.5022

**Table 5:** List of all reagents used in the study.

Substrate	Producer	Purity Degree
N-isopropylacrylamide	Sigma-Aldrich (Darmstadt, Germany)	Reagent grade
N,N′-methylenebisacrylamide	Sigma-Aldrich (Darmstadt, Germany)	Reagent grade
Gum arabic	POCH S.A. (Gliwice, Poland)	Reagent grade
Sodium alginate	Sigma-Aldrich (Darmstadt, Germany)	Reagent grade
Poly(vinyl alcohol) (M_w_ = 72,000 g/mol)	POCH S.A. (Gliwice, Poland)	Reagent grade
Aloe vera lyophilisate	Zrób sobie krem (Prochowice, Poland)	n.d.
Glycerin	POCH S.A. (Gliwice, Poland)	Reagent grade
Poly(ethylene glycol) diacrylate (PEGDA) (M_n_ = 700 g/mol)	Sigma-Aldrich (Darmstadt, Germany)	Reagent grade
Ammonium persulphate	POCH S.A. (Gliwice, Poland)	Reagent grade
Salicylic acid	Sigma-Aldrich (Darmstadt, Germany)	Reagent grade
Fluocinolone acetonide	Sigma-Aldrich (Darmstadt, Germany)	Reagent grade
Buffer solutions with pH values of 4.0, 7.4, and 9.0	Chempur (Piekary Śląskie, Poland)	n.d.
Ethyl alcohol (96%, *v*/*v*)	Fisher Scientific (Hampton, NH, USA)	Reagent grade
MEM with Earle’s Salts, with Stable Glutamine	Biowest (Nuaillé, France)	Reagent grade
Fetal Bovine Serum	Biowest (Nuaillé, France)	Reagent grade
Antibiotic Antimycotic Solution (100×)	Sigma-Aldrich (Darmstadt, Germany)	Reagent grade
MEM Non-Essential Amino Acids Solution (100×)	Gibco, Life Technologies (Waltham, MA, USA)	Reagent grade
CytoTox-Fluor™ Cytotoxicity Assay	Promega (Madison, WI, USA)	Reagent grade

## Data Availability

The data that support the findings of this study are contained within the article.
